# Supplementary material for: Present and Future of IgA Nephropathy and Membranous Nephropathy Immune Monitoring: Insights from Molecular Studies
Source: Int J Mol Sci. 2023 Aug 23;24(17):13134. doi: 10.3390/ijms241713134 (PMC10487514; doi:10.3390/ijms241713134)
Supplement: Supplementary file 1 [file ijms-24-13134-s001.zip › ijms-2517709-supplementary.pdf]

Study selection criteria

To conduct this narrative review, we mainly focused our search on biomarkers that are related to disease pathogenic mechanisms, and we discussed the use of omics in patient risk stratification and clinical guidance. Our literature search has been performed accordingly by using specific key words related to each section, which are listed below:

- Biomarkers AND/OR immune monitoring, antibodies, complement, immune cells, genetic risk, RNA sequencing, transcriptomics, proteomics, prognosis, predictors.

We selected studies published within the last 10 years with the following inclusion criteria:

Type of biomarkers investigated:

- Biomarkers biologically plausible according to disease pathogenesis
- Biomarkers investigated with omics: genomics, epigenomics, transcriptomics, proteomics

Measures of outcome assessed:

- Prognosis (expressed as change in proteinuria, serum creatinine, and/or eGFR, ESKD)
- Response to therapy (expressed as reduction in proteinuria and/or serum creatinine after a complete course of therapy)
- Disease diagnosis (especially for potential new autoantibodies in MN)
- Post-diagnosis MN relapse (expressed as significant increase in proteinuria)
- Post-transplant disease recurrence

After selecting the studies according to the aforementioned criteria, we mainly focused our discussion on biomarkers with high level of evidence (i.e. investigated in large-scale studies, investigated in different ethnicities, validated by multiple cohorts) and/or novelty (i.e. investigated with relatively new techniques/methods)
